# Yeast interfering RNA larvicides targeting neural genes induce high rates of *Anopheles* larval mortality

**DOI:** 10.1186/s12936-017-2112-5

**Published:** 2017-11-13

**Authors:** Keshava Mysore, Limb K. Hapairai, Longhua Sun, Elizabeth I. Harper, Yingying Chen, Kathleen K. Eggleson, Jacob S. Realey, Nicholas D. Scheel, David W. Severson, Na Wei, Molly Duman-Scheel

**Affiliations:** 1 Dept. of Medical and Molecular Genetics, Indiana University School of Medicine, 1234 Notre Dame Avenue, South Bend, IN 46530 USA; 2 The University of Notre Dame, Eck Institute for Global Health, Notre Dame, IN 46556 USA; 3 Dept. of Biological Sciences, The University of Notre Dame, Notre Dame, IN 46556 USA; 4 Dept. of Civil and Environmental Engineering and Earth Sciences, The University of Notre Dame, Notre Dame, IN 46556 USA; 5 Dept. of Medicine, Indiana University School of Medicine, 1234 Notre Dame Avenue, South Bend, IN 46530 USA

**Keywords:** Mosquito, Vector, Malaria, RNAi, *Saccharomyces cerevisiae*, Larviciding, Pesticide, Synapse, Brain, Larvae

## Abstract

**Background:**

Although larviciding can reduce the number of outdoor biting malaria vector mosquitoes, which may help to prevent residual malaria transmission, the current larvicide repertoire is faced with great challenges to sustainability. The identification of new effective, economical, and biorational larvicides could facilitate maintenance and expansion of the practice of larviciding in integrated malaria vector mosquito control programmes. Interfering RNA molecules represent a novel class of larvicides with untapped potential for sustainable mosquito control. This investigation tested the hypothesis that short interfering RNA molecules can be used as mosquito larvicides.

**Results:**

A small interfering RNA (siRNA) screen for larval lethal genes identified siRNAs corresponding to the *Anopheles gambiae suppressor of actin* (*Sac1*), *leukocyte receptor complex member* (*lrc*), and *offtrack* (*otk*) genes. *Saccharomyces cerevisiae* (baker’s yeast) was engineered to produce short hairpin RNAs (shRNAs) for silencing of these genes. Feeding larvae with the engineered yeasts resulted in silenced target gene expression, a severe loss of neural synapses in the larval brain, and high levels of larval mortality. The larvicidal activities of yeast interfering RNA larvicides were retained following heat inactivation and drying of the yeast into user-friendly tablet formulations that induced up to 100% larval mortality in laboratory trials.

**Conclusions:**

Ready-to-use dried inactivated yeast interfering RNA larvicide tablets may someday be an effective and inexpensive addition to malaria mosquito control programmes and a valuable, biorational tool for addressing residual malaria transmission.

**Electronic supplementary material:**

The online version of this article (10.1186/s12936-017-2112-5) contains supplementary material, which is available to authorized users.

## Background

Although insecticide-treated nets (ITNs) and indoor residual spraying (IRS) have been the backbone for malaria vector control, resistance to insecticides used for these applications is a growing problem. Furthermore, these interventions cannot control mosquitoes that bite or rest outdoors [1]. As residual transmission of malaria becomes increasingly problematic, there is renewed interest in the use of larval source management (LSM) for reduction of residual transmission. In the first half of the twentieth century, LSM was a large-scale and highly effective method for control of malaria. Although large-scale LSM programmes were disbanded and replaced by IRS with dichlorodiphenyltrichloroethane (DDT) in the latter part of the century, LSM is still a major component of integrated mosquito control programmes in some parts of the world [2]. Moreover, LSM, unlike IRS and ITNs, can reduce the number of mosquitoes that enter houses and the number of outdoor-biting mosquitoes [3].

Larviciding, a form of LSM, involves the application of chemical or biological agents to water bodies for targeting of immature aquatic larvae and pupae before they become malaria vector mosquitoes. Integrated control programmes that included larviciding were successful in Tanzania, Sudan, and Mauritius [2, 3], and a recent study in Kenya demonstrated that long-lasting FourStar™ briquette larvicides significantly reduced mean densities of indoor- and outdoor-biting adult malaria vector mosquitoes [4]. The World Health Organization (WHO) recommended that larviciding, when used as a supplement to ITNs and IRS in sub-Saharan Africa, is cost-effective for malaria control in urban settings where vector breeding sites are few, fixed, and findable [2]. However, given the increase of reported insecticide resistance and rising concern for the negative effects of pesticides on non-target organisms, the current pesticide repertoire is faced with great challenges to sustainability [1]. The identification of new, environmentally safe, cost-effective larvicides is critical if the current levels of larviciding are to be sustained or expanded.

Interfering RNA molecules represent a novel class of larvicides with untapped potential for sustainable mosquito control. Although RNA interference (RNAi) is beginning to attract attention in agricultural biotechnology communities [5–7], RNAi is still a largely unexplored approach for control of disease vector mosquitoes. The RNAi pathway is initiated by Dicer, which cleaves long pieces of double stranded RNA (dsRNA) into small interfering RNAs (siRNAs) which silence genes that are complementary in sequence. Most mosquito researchers use longer (300–400 bp) dsRNA molecules for RNAi. However, the short length (21–25 bp) of custom siRNAs and their short hairpin RNA (shRNA) counterparts facilitates the design of interfering RNA that targets mosquitoes, but not humans or other non-target organisms [8, 9]. siRNAs have facilitated our functional genetic characterization of mosquito larval development [10–14]. This investigation tested the hypothesis that short interfering RNA molecules can be utilized as larvicidal agents for control of *Anopheles* mosquitoes. The authors recently conducted a high throughput screen (MDS, in preparation) that resulted in the identification of > 100 mosquito siRNA larvicides. Here, the characterization of highly toxic interfering RNAs targeting three larval lethal mosquito genes identified in the screen, *suppressor of actin* (*Sac1*), *leukocyte receptor complex member* (*lrc*), and *offtrack* (*otk*), is described. The study then describes the bioengineering and testing of a *Saccharomyces cerevisiae* (baker’s yeast) system for delivery of these interfering RNA molecules to *Anopheles gambiae* mosquito larvae. The results of this investigation demonstrate that heat-inactivated dry yeast interfering RNA pellets targeting these genes induce severe defects in the mosquito central nervous system and up to 100% larval mortality.

## Methods

### Animal rearing

The *A. gambiae M* strain (obtained from N. Besansky) and the *Aedes aegypti* Liverpool-IB12 (LVP-IB12; obtained from D. Severson) strain were used in these studies and maintained generally as described [15], except that an artificial membrane feeding system was used for delivery of sheep blood (HemoStat Laboratories, Dixon, CA) to adult female mosquitoes. The insects were reared in an insectary maintained at 26 °C, at ~ 80% humidity, and under a 12 h light/12 h dark cycle with 1 h crepuscular periods at the beginning and end of each light cycle.

### Identification of larvicidal siRNAs

An siRNA larvicide screen led to the identification of siRNAs Sac1.1 and Sac1.91 which correspond to *Sac1* (*AGAP000891*), lrc.2 and lrc.51 which target *lrc* (*AGAP008903*), and otk.16 and otk.94 which correspond to *otk* (*AGAP011489*). For the screen, custom siRNAs corresponding to *Anopheles* orthologs of a subset of *Drosophila* larval lethal genes [16] as well as a control siRNA [14] with no known target in *An. gambiae* were purchased from Integrated DNA Technologies. The sequences targeted by these siRNAs, are as follows:

Sac1.1: 5′CCAACUGCAUCGACUGUCUGGACCG3′

Sac1.91: 5′GCCUAAUCAACCUGAUCGACCACAA3′

lrc.2: 5′AUUGGUUCAUCGAGCGUGAACGCAA3′

lrc.51: 5′CCAGCACCAGCCAACGAGGAACACU3′

otk.16: 5′GCUCGGUACGGUACAGUUUCACUGC3′

otk.94: 5′GGACAAAGAUCUGCAGUAUCUGCAU3′

Control: 5′GAAGAGCACUGAUAGAUGUUAGCGU3′

Basic local alignment search tool (BLAST) [17] searches indicated that the siRNAs corresponding to *Sac1*, *lrc*, and otk lacked identical matches in *Aedes* mosquitoes, amphibians, birds, fish, fungi, humans, mammals, plants, and reptiles (Additional file 1). The screen involved a dual-testing approach that included microinjection of third instar larvae as well as soaking of first instar larvae, both of which were performed in duplicate. For injections, ~ 10 pmol custom screening siRNA were injected in a 30 nL volume per larva (n = 30/condition/replicate) using previously described methodology [18, 19]. siRNAs were simultaneously screened through delivery via larval soaking (per [20]), which permitted a second round of selection to identify siRNAs that are capable of killing mosquitoes early in larval development following only brief exposure to siRNA, which may better simulate field conditions. For soaking experiments, 20 first instar larvae/condition/replicate were exposed to 20 µL of 0.5 µg/µL siRNA for 4 h. Following siRNA treatment, larvae were reared and lethality evaluated per the WHO [21] larvicide testing guidelines. The mortality of larvae treated with siRNA via soaking or microinjection was compared to control-treated animals using the Fisher’s exact test [22]. In the larval screen, as well as all other larvicide assays reported here, larvae that were not treated with larvicidal or control RNA and fed a normal laboratory diet were reared in parallel. No significant differences in the mortality of control-treated vs. non-treated larvae were detected in any of the assays conducted in this investigation. The results for control-treated larvae are reported herein.

### Bacteria interfering RNA larvicide preparation and feeding

Heat-killed non-pathogenic *Escherichia coli* expressing dsRNAs of interest were constructed and fed to larvae beginning in the first instar (L1) stage using the general protocol of Whyard et al. [20], which was adapted for use in *An. gambiae.* Strain *HT115*-*DE3*, obtained from the *Caenorhabditis* Genetics Center (which is funded by the NIH Office of Research Infrastructure Programs, P40 OD010440), was used in these studies. Bacteria were transformed with the dsRNA transcription plasmid pL4440 (deposited at Addgene by Andrew Fire; plasmid # 1654), which contains forward and reverse T7 polymerase binding sites that flank a multiple cloning site in which DNA corresponding to the Sac1.1, lrc.2, or otk.16 siRNA target sequences had been cloned. This permitted inducible expression of dsRNA in *E. coli*, which were prepared and fed to larvae as discussed [23], with liver powder being substituted for ground fish food (Doctors Foster and Smith, Stable Diet, Rhinelander, Wisconsin). *Escherichia coli* that had been transformed with GFP:L4440, which was deposited at Addgene by Guy Caldwell (Addgene plasmid # 11335) and expresses dsRNA corresponding to GFP, was used as a control feeding strain in these experiments. Data were compiled from at least two biological replicate experiments, each with four replicate cups bearing 20 animals for each treatment (n = 240 control-treated larvae, 240 lrc.2-treated larvae, and 160 otk.16-treated larvae) and assessed by ANOVA with Tukey’s multiple comparison test.

### Yeast interfering RNA larvicide preparation and feeding

shRNA-encoding DNA oligonucleotides corresponding to the Sar1.1, lrc.51, otk.16, and control siRNA target sequences were custom synthesized by Invitrogen Life Technologies. These shRNA expression cassettes were cloned into the non-integrating *pRS426 GPD* yeast shuttle vector, which has a *URA3* marker and permits constitutive expression of inserts cloned downstream of a *GPD* promoter [24]. Following sequencing to confirm the hairpin expression cassette sequences, the plasmids were transformed into *S. cerevisiae* strain *BY4742* [25] (genotype *MATα his3Δ1 leu2Δ0 lys2Δ0 ura3Δ0*). Transformants were selected by checking for growth on minimal media lacking uracil. Following selection, the yeast was grown to an OD_600_ of 3.0 under standard conditions in synthetic media. For gel-coated formulations, the Whyard et al. [23] procedure for bacterial pellet preparation was used in conjunction with a 50 mL yeast culture. For preparation of dried tablet formulations, 50 mL of yeast culture was transferred to 50 mL conical tubes and pelleted by centrifugation for 20 min at 4000 rpm (Eppendorf 5810R plus), and the supernatant (culture media) was discarded. For heat inactivation, the pellet was placed in a 70 °C water bath for 5 min. The yeast pellet was then placed into a 2 mL tube which contained 10 mg of fish food and centrifuged for 1 min at ~ 13.2 rpm (Eppendorf, 5415D). The supernatant was discarded, and the open tubes were placed in an incubator at 30 °C for 48 h to permit evaporation of the remaining media. The dried tablets were stored in capped microfuge tubes at – 20 °C. The final weight of each yeast tablet averaged 95 mg, including 10 mg of fish food and 85 mg of yeast (~ 1.6 × 10^10^ cells).

For each replicate, agarose-gel coated yeast pellets were divided into three portions, with each portion fed daily to 20 larvae beginning in L1 for 3 days. For dried yeast formulations, at the beginning of each experiment, one control or experimental tablet was placed in a container with 20 L1 animals. The larval bioassays were performed in 500 mL plastic cups containing 50 mL of water. For the gel-coated live yeast experiments, data were compiled from two biological replicate experiments, each with three replicates per condition and 20 larvae per replicate for a total n of 120 larvae/condition. For the gel-coated inactivated yeast experiments, data were compiled from three biological replicate experiments, each with three replicates per condition and 20 larvae per replicate for a total n of 180 larvae/condition. For the dried inactivated yeast tablet experiments, data were compiled from three biological replicate experiments with four replicates per condition and 20 animals per replicate (n = 240 larvae/condition). Yeast interfering RNA larvicide data were assessed by ANOVA with Tukey’s multiple comparison test.

### Whole mount in situ hybridization and immunohistochemistry

Riboprobes corresponding to the *Sac1*, *lrc*, and *otk* genes were synthesized according to the Patel [26] protocol, and in situ hybridization experiments were performed in duplicate according to the Haugen et al. [27] protocol. At the initiation of tissue fixation, larvae were living. Stained tissues were mounted, analysed, and imaged using a Zeiss Axioimager equipped with a Spot Flex camera. For quantification of transcripts, mean gray values were calculated for digoxigenin-labeled transcript signal in control or experimental brains. For each probe, the results from three biological replicate experiments were compiled (n = 40 control-treated brains/per probe, n = 85 Sac1.1-treated brains, n = 80 lrc.51-treated brains, and n = 80 otk.16-treated brains). A t test was used to analyse transcript quantification data.

Immunohistochemical staining experiments were performed as described [28, 29] using the nuclear stain TO-PRO-3 iodide (Molecular Probes, Eugene, OR) and mAb 3C11, which recognizes the Synapsin-1 cleaved fragment [30]. mAb 3C11 was deposited by E. Buchner at the Developmental Studies Hybridoma Bank, a facility created by the NICHD of the NIH and maintained at The University of Iowa. Larvae were still living at the initiation of tissue fixation. Following processing, the tissues were mounted and then imaged at the IUSM Flow Cytometry and Imaging Core facility using a Zeiss 710 confocal microscope and Zen software. FIJI ImageJ and Adobe Photoshop CC 2014 software were used to analyse the images. Three biological replicate experiments were performed, with a total of 25 brains evaluated per condition.

## Results

### *Sac1*, *otk* and *lrc* are mosquito larval lethal genes

**Fig. 1:**
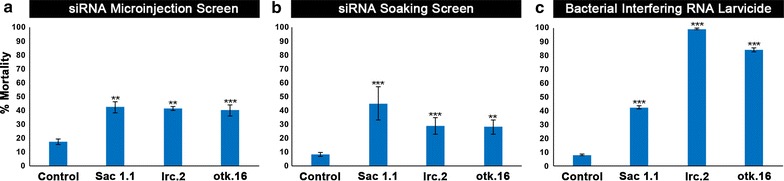
Larval mortality is induced by interfering RNA larvicides with target sites in the *Sac1*, *lrc*, and *otk* genes. The Sac1.1, lrc.2, and otk.16 siRNAs were identified in a screen for larval lethal genes in which siRNAs were evaluated by microinjection of third instar larvae (**a**) and through brief soaking treatment of first instar larvae (**b**). In the screen, an siRNA with no known target in *Anopheles gambiae* served as the control (**a**, **b**). The screen was performed in duplicate (see “Methods” for further details). For each replicate, 30 animals per treatment were microinjected, and 20 animals/treatment were soaked. Data were analysed with Fisher’s exact test [22]. **c** The larvicidal activity of these interfering RNAs was further confirmed when significant mortality was observed in larvae fed with heat-inactivated *E. coli* expressing dsRNA corresponding to the Sac1.1, lrc.2, and otk.16 target sites. Animals fed with bacteria expressing dsRNA corresponding to GFP served as the control in these experiments. Data were compiled from replicate experiments (n = 240 control-treated larvae, 160 Sac1.1-treated larvae, 240 lrc.2-treated larvae, and 160 otk.16-treated larvae) and assessed by two-way ANOVA with Tukey’s multiple comparison test. ***p < 0.001 in comparison to control-fed larvae; **p < 0.05 in comparison to control-fed larvae; error bars denote standard errors of the mean (SEMs)

A dual-screening approach was employed to evaluate siRNAs corresponding to *Anopheles* orthologs of *Drosophila melanogaster* larval lethal genes [16] for larvicidal activity. siRNAs were screened both through microinjection of third instar larvae (Fig. 1a) as well as through 4 h soaking treatment of first instar larvae (Fig. 1b). Microinjection of siRNA Sac1.1, which corresponds to the *Sac1* gene, resulted in 42 ± 4% larval mortality (Fig. 1a; p < 0.01 vs. control siRNA injected animals). siRNA lrc.2, which targets a sequence in the *lrc* gene, resulted in significant 42 ± 2% mortality following injection (Fig. 1b; p < 0.05 vs. control), while injection of siRNA otk.16, which corresponds to *otk*, yielded 40 ± 4% larval mortality (p < 0.001 vs. control). siRNAs Sac1.1, lrc.2, and otk.16 also displayed significant larvicidal activity (p < 0.01 vs. control) in short-term exposure soaking experiments conducted with first instar larvae, inducing 45 ± 12, 29 ± 6 and 28 ± 5% larval mortality, respectively (Fig. 1b). These experiments indicated that exposure to the siRNAs either early (L1) or later (L3) in larval development can result in death.

The larvicidal activity of these interfering RNAs was further confirmed by feeding larvae heat-killed *E. coli* prepared as described by Whyard et al. [23] that had been engineered to express dsRNA corresponding to the Sac1.1, lrc.2, and otk.16 siRNA target sequences. These experiments, in which bacteria interfering RNA larvicides Sac1.1, lrc.2, and otk.16 induced 43 ± 1, 99 ± 0, and 84 ± 1% death (Fig. 1c), further demonstrated the larvicidal capacity of these interfering RNA molecules. Silencing experiments were also conducted with siRNAs corresponding to additional target sequences in the *Sac1*, *lrc*, and *otk* genes. Injection of siRNA Sac1.91, which corresponds to an alternative target site in *Sac1*, induced 29 ± 2% larval mortality (p < 0.05 vs. control siRNA). siRNA lrc.51, which targets an alternate site in *lrc*, resulted in 55 ± 1% death (p < 0.001 vs. control siRNA) following soaking treatment. Injection of siRNA otk.94, which corresponds to an alternative target site in *otk*, induced 40 ± 10% mortality (p < 0.001 vs. control siRNA). These results indicate that targeting alternative sites in *Sac1*, *lrc*, or *otk* induces larval death, providing further evidence that these genes are required for larval survival.

### Exploration of yeast interfering RNA delivery systems

**Fig. 2:**
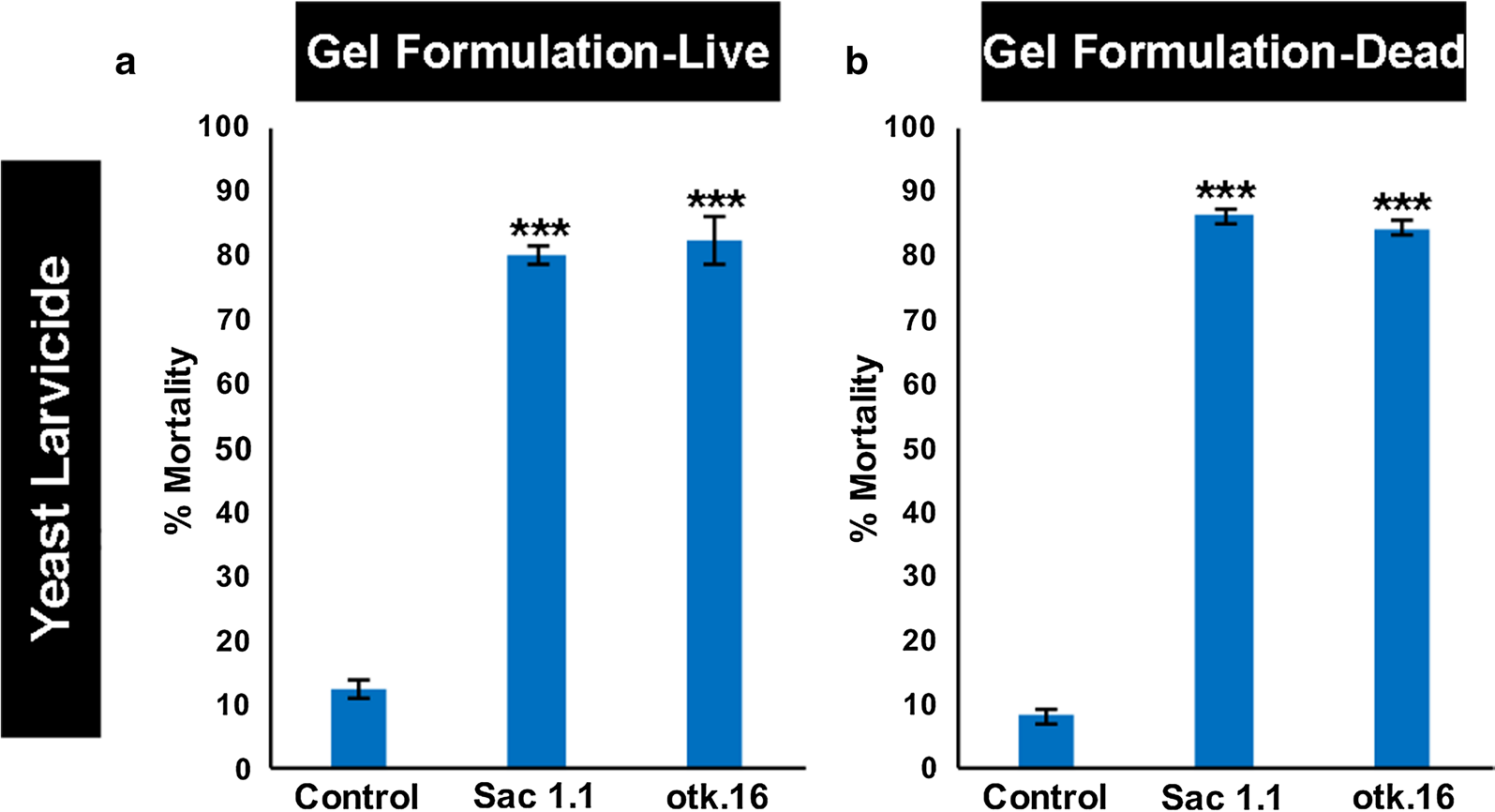
Yeast interfering RNA larvicides induce significant mortality. Significant larval death was observed in larvae fed with gel-coated live (**a**) or heat-inactivated (**b**) yeast engineered to express shRNA corresponding to the Sac1.1 and otk.16 siRNA target sequences (as compared to control yeast expressing shRNA corresponding to the control siRNA sequence). Data were compiled from replicate experiments, with 120 larvae evaluated per condition for live yeast and 180 larvae assessed per condition for inactive yeast (see “Methods” for details). Data were statistically assessed by ANOVA with Tukey’s multiple comparison test. No statistically significant differences were observed in the larvicidal capacity of live vs. heat-inactivated gel-coated yeast interfering RNA larvicides. ***p < 0.001 in comparison to control-fed larvae; error bars denote SEMs

The results from the siRNA larval lethal screen and bacterial short dsRNA larvicide studies supported the hypothesis that yeast expressing shRNA that targets larval lethal genes could be used as larvicides. In initial experiments, shRNA corresponding to the Sac1.1, otk.16, or control siRNA target sequence was constitutively expressed from a non-integrating multi-copy yeast shuttle plasmid that was transformed into *S. cerevisiae*. This yeast strain, as well as a control yeast strain expressing shRNA with no known target in *An. gambiae*, was initially fed to larvae in an agarose gel-covered formulation (Fig. 2a) using methodology that was comparable to the preparation of bacterial interfering RNA larvicides. Since both live and heat-inactivated bacteria can induce mosquito death [23], both live and dead yeast covered in agarose were tested. While control-fed larvae survived, gel-coated live yeast larvicides Sac1.1 and otk.16 killed 80 ± 4 (Fig. 2a; p < 0.001 vs. control) and 83 ± 4% of mosquitoes (Fig. 2a; p < 0.001 vs. control), respectively. Heat-inactivated gel-coated yeast Sac1.1 induced 86 ± 1% mortality (Fig. 2b; p < 0.001 vs. control), while heat-inactivated gel-coated yeast otk.16 induced 84 ± 1% mortality (Fig. 2b; p < 0.001 vs. control). No significant differences were observed in the larvicidal capacity of live vs. heat-inactivated yeast interfering RNA larvicides (Fig. 2a vs. b). Heat-inactivated Sac1.1 and otk.16 yeast fed to *A. aegypti* larvae, in which the target sequences of these interfering RNAs are not conserved, did not induce larval death (Additional file 2; p = 0.66).

### Dried inactivated yeast interfering RNA larvicide tablets induce significant larval death

**Fig. 3:**
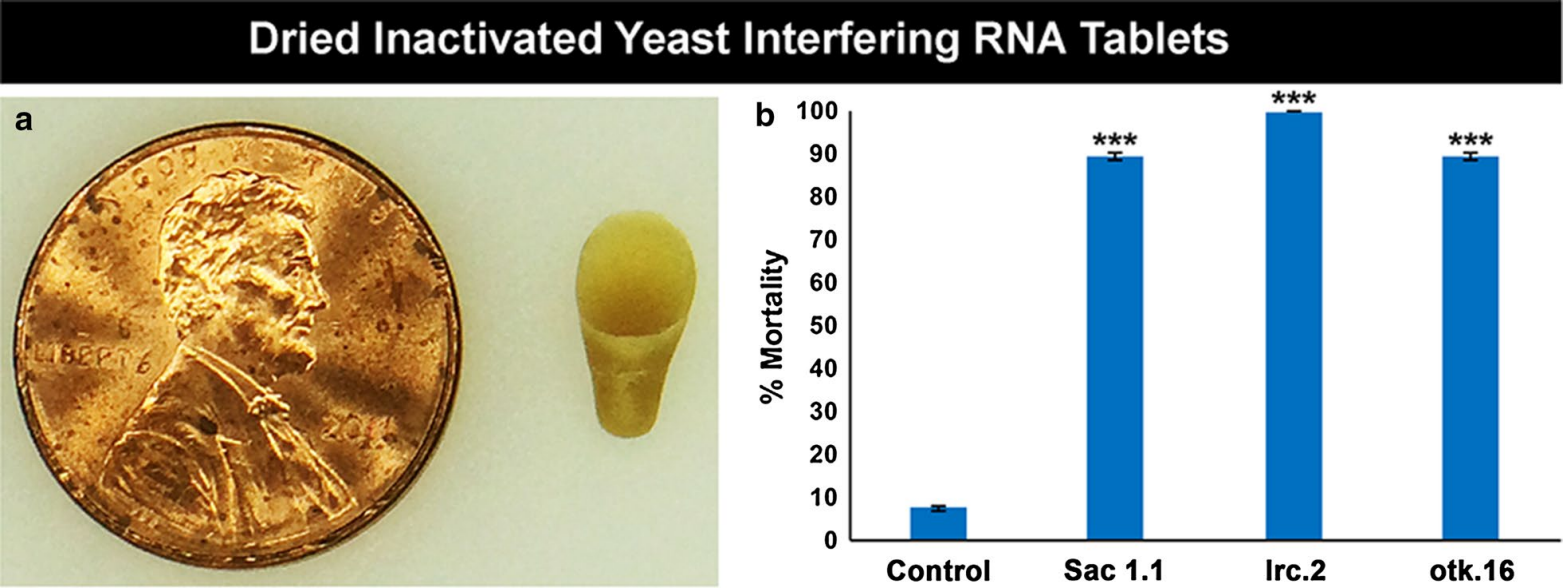
Dried inactivated yeast interfering RNA tablets induce significant larval death. Dried inactivated yeast interfering RNA larvicide tablets (**a**; penny shown for scale) were prepared and fed to 20 larvae. Significant larval death was observed in larvae fed with yeast expressing shRNA hairpins corresponding to the Sac1.1, lrc.51, and otk.16 target sequences as compared to larvae fed control yeast interfering RNA tablets (**b**). Data were compiled from three biological replicate experiments (total n = 240 larvae/condition; see “Methods” for details) and analysed by ANOVA with Tukey’s multiple comparison test. ***p < 0.001 in comparison to control-fed larvae; error bars denote SEMs

Although agarose-coated yeast larvicides induced high mortality rates (Fig. 2), these formulations are sticky and non user-friendly. Also, the water in containers treated with the gel-coated yeast becomes cloudy, which may not be desired by prospective users. To circumvent these issues, dried tablets of inactivated yeast (Fig. 3a), which are comparable in appearance to yeast nutritional tablets sold as human dietary supplements, were therefore prepared and evaluated. This formulation is easier to handle, does not cloud the water in treated containers, and is readily consumed by *Anopheles* larvae. Treatment of 50 mL water containing 20 larvae with a 95 mg dried tablet of yeast interfering RNA larvicide Sac1.1 induced 89 ± 1% larval mortality (Fig. 3b; p < 0.001 vs. control). Dried yeast interfering RNA larvicide otk.16 induced 89 ± 1% larval mortality (Fig. 3b; p < 0.001 vs. control). Yeast expressing shRNA corresponding to the lrc.51 target sequence was also prepared, and these tablets induced 100 ± 0% larval mortality (Fig. 3b; p < 0.001 vs. control). These results demonstrated that dried inactivated yeast interfering RNA larvicide tablets have highly significant larvicidal activity.

### Yeast interfering RNA larvicide-mediated silencing of *Sac1*, *otk*, or *lrc* disrupts neural synapses in the larval brain

**Fig. 4:**
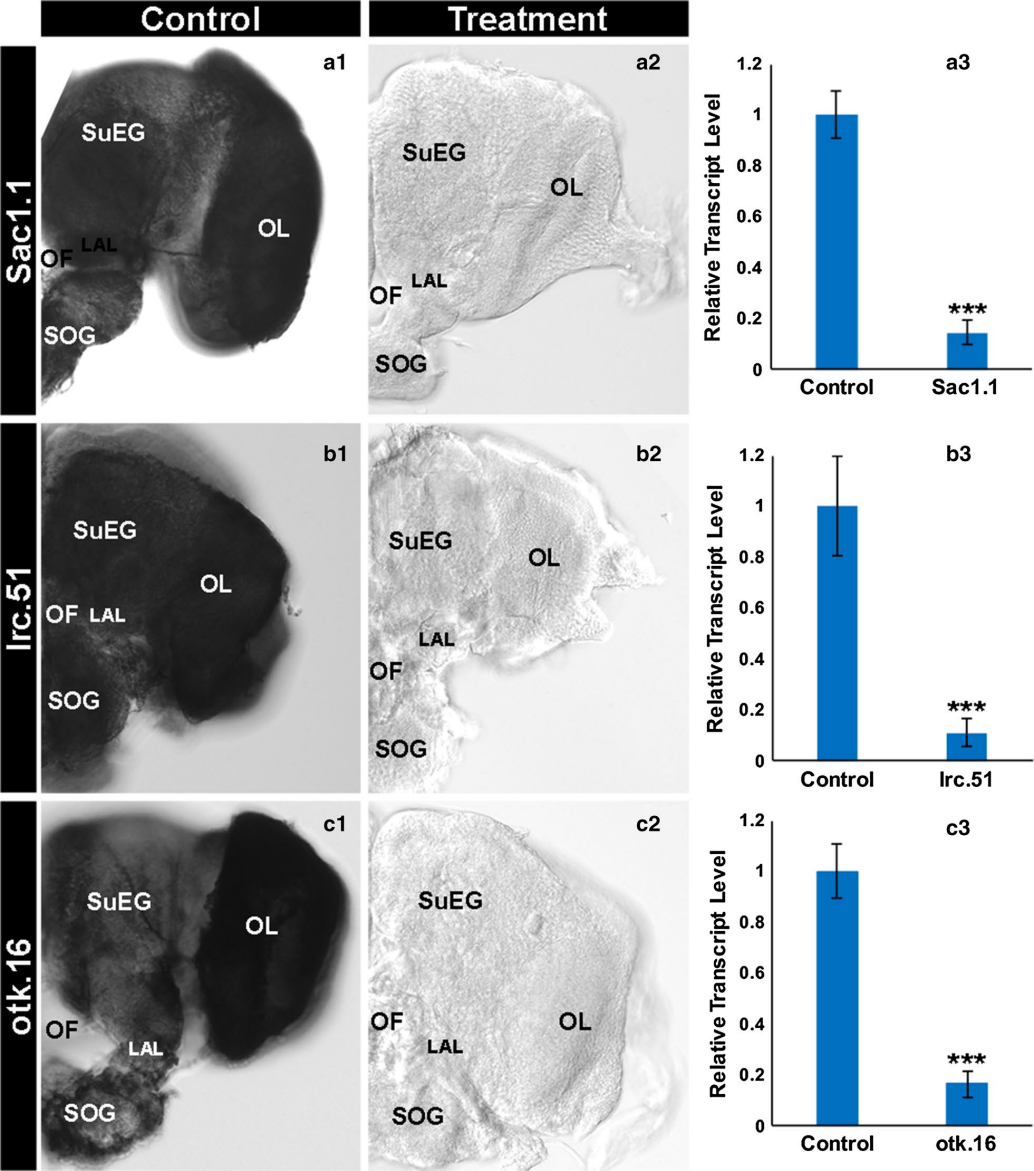
Confirmed silencing of the *Sac1*, *lrc*, and *otk* genes in the larval brain by dried inactivated yeast interfering RNA tablets. Significantly lower levels of *Sac1* (**a1**–**a3**), *lrc* (**b1**–**b3**), and *otk* (**c1**–**c3**) transcripts were detected in the L4 brains of larvae fed with the Sac1.1 (**a2**), lrc.51 (**b2**), and otk.16 (**c2**) dried inactivated yeast interfering RNA larvicides vs. animals fed with control yeast (**a1**, **b1**, **c1**). For each probe, results from three biological replicate experiments were compiled (n = 85 Sac1.1-treated brains, n = 80 lrc.51-treated brains, and n = 80 otk.16-treated brains; n = 40 control-treated brains/per experiment). Data were evaluated by t test (***p < 0.001 in comparison to control-fed larvae). The brains are oriented dorsal upward in this figure. LAL, larval antennal lobe; OF, olfactory foramen; OL, optic lobe; SOG, sub-oesophageal ganglion; SuEG, supra-oesophageal ganglion

In *D. melanogaster*, *Sac1* encodes phosphatidylinositol phosphatase, which functions to dephosphorylate phosphatidylinositol 4-phosphate for generation of phosphatidylinositol and acts as an axon guidance regulator [31]. The *D. melanogaster otk* gene encodes a member of the Tyrosine protein kinase superfamily which functions as a neural cell adhesion molecule to regulate axon guidance [32, 33]. The *D. melanogaster CG6700* gene is an *lrc* ortholog and encodes a conserved C-terminal SAC3/GANP domain-containing protein member of the dystroglycan–dystrophin complex [34–36]. *CG6700* was listed in a catalog of genes likely to function in synapse assembly and function [37], but its function in this capacity remained to be tested. Given the reported neural functions of the *Sac1*, *lrc*, and *otk Drosophila* orthologs, the functions of these genes were assessed in the mosquito brain (Fig. 4). Detection (Fig. 4a2 vs. a1) and quantification (Fig. 4a3) of *Sac1* transcript levels in the L4 larval brain demonstrated that ingestion of yeast interfering larvicide Sac1.1 resulted in 85 ± 5% reduction of *Sac1* transcripts (p < 0.001). Detection (Fig. 4b2 vs. b1) and quantification (Fig. 4b3) of L4 larval brain *lrc* transcripts showed that ingestion of yeast interfering RNA larvicide lrc.51 resulted in 88 ± 6% reduction of *lrc* transcripts. Likewise, detection (Fig. 4c2 vs. c1) and quantification (Fig. 4c3) of *otk* transcripts in the L4 larval brain indicated that ingestion of yeast interfering RNA larvicide otk.16 resulted in 83 ± 5% reduction of *otk* transcripts.

**Fig. 5:**
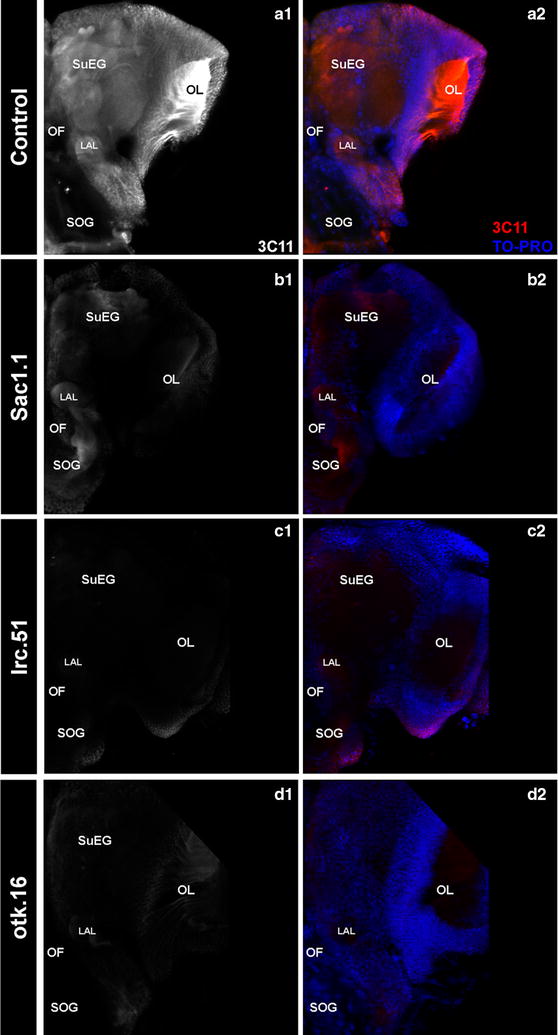
Neural defects observed in larvae treated with yeast interfering RNA larvicides Sac1.1, lrc.51, and otk.16. L4 larval brains were labeled with mAb3C11 (white in **a1**–**d1**; red in **a2**–**d2**), which labels expression of Synapsin, a marker for the neuropil and synaptic active zones. TO-PRO was used to counter-stain nuclei in the brain (blue in **a2**–**d2**). The brains of larvae fed with yeast expressing shRNAs Sac1.1 (**b1**, **b2**), lrc.51 (**c1**, **c2**), and otk.16 (**d1**, **d2**) yeast interfering RNA tablets show loss of staining in the synaptic neuropil regions when compared with animals fed with control yeast (**a1**, **a2**). Three biological replicate experiments were performed. The data shown are representative of the results from 25 brains evaluated per condition. LAL, larval antennal lobe; OF, olfactory foramen; OL, optic lobe; SOG, sub-oesophageal ganglion; SuEG, supra-oesophageal ganglion

Detection of Synapsin expression (with mAb 3C11 [30]), a marker of active neural synapses in multiple arthropod species [10], was used to assess synaptic activity in the brains of animals fed with these yeast interfering RNA larvicides. Synapsin expression was severely reduced in the synaptic neuropiles of animals fed with yeast interfering RNA larvicides Sac1.1 (Fig. 5b1, b2 vs. control-fed animals in a1, a2), lrc.51 (Fig. 5c1, c2 vs. a1, a2), and otk.16 (Fig. 5d1, d2 vs. a1, a2). These severe neural phenotypes are likely to be primary causes of larval death in animals fed with yeast interfering RNA larvicides targeting these genes.

## Discussion

Numerous studies have demonstrated that target gene silencing through RNAi can promote insect death [6]. For example, Baum et al. [38] engineered transgenic corn plants expressing dsRNA targeting the western corn rootworm *Diabrotica virgifera virgifera*. dsRNA insecticidal corn targeting this species was recently registered by the US Environmental Protection Agency [39], which deemed that this intervention meets the regulatory standards described in the Federal Insecticide, Fungicide, and Rodenticide Act [40]. Likewise, Whyard et al. [41] showed that *Tribolium castaneum*, *Acyrthosiphon pisum*, and *Manduca sexta* were killed when fed species-specific dsRNA targeting *vATPase.* The sequence specificity of RNAi facilitated their design of dsRNAs that targeted specific chosen insects, but which did not kill non-target insects, including closely related insect species [41]. Similarly, this study designed yeast interfering RNA larvicides with target sequences in *Anopheles* larval lethal genes that are not perfectly conserved in other organisms (Additional file 1) and which failed to kill *A. aegypti* (Additional file 2). Of course, the ability of an siRNA to have off-target effects is complicated [42]. It is difficult to predict, on the basis of sequence information alone, whether these yeast interfering RNA larvicides could impact gene expression in non-target organisms [42], and it will be important to conduct more detailed toxicity studies on these larvicides. However, in general, interfering RNA pesticides are believed to be much safer than chemical pesticides [43]. MonSanto [43] concluded, in an extensive evaluation of RNA molecules prepared for the United States Environmental Protection Agency, that RNA based products are an important technology with great promise for pest mitigation applications while presenting an overwhelmingly desirable safety profile, particularly when compared to conventional pesticides. Moreover, although many had questioned whether interfering RNAs were sufficiently stable for field applications, dsRNA stability has actually been reported to be quite high [44, 45]. These and other studies suggest that RNAi can be exploited to control insect agricultural pests.

RNAi has been used extensively for functional analysis of mosquito genes [10–14]. However, RNAi has not yet emerged as a method for mosquito control. The results of this investigation, in which a number of larval lethal genes to be targeted and an effective delivery system for interfering RNAs targeting these genes were identified, suggest that the addition of RNAi pesticides to integrated mosquito control programmes may be feasible and beneficial. Through the identification of multiple larval lethal genes and target sites in these genes, including those described here and additional RNAi targets in the *An. gambiae* genome that have been discovered and tested, the authors hope to build an arsenal of interfering RNAs that can be used to combat resistance to traditional chemical pesticides. This arsenal can also be used to combat future resistance that might develop to any single interfering RNA pesticide.

The broad application of RNAi for pest control is dependent upon production of dsRNA in an economically feasible, scalable, and sustainable fashion. dsRNA manufacture has traditionally relied on expensive, carbon-intensive chemical synthesis, resulting in high costs [5, 6] and a perception that use of RNAi for mosquito control would be prohibitively expensive. Additionally, a perceived high operational cost and the complexity of *Anopheles* larviciding in general has deterred some mosquito control programmes from adopting LSM strategies for malaria vector control [3]. The use of ready-to-use yeast interfering RNA larvicide tablets, which are a user-friendly economically feasible biorational alternative to conventional larvicides may help to address these concerns. While *Pichia pastoris* has been used for expression of recombinant DNA and dsRNA to target *Aedes* [46, 47], in this investigation, we opted to use *S. cerevisiae*, for which many mutant strains and plasmid constructs exist. Interfering RNA was affordably propagated through cultivation of the yeast. The first three *Anopheles* interfering RNA larvicides generated in *S. cerevisiae*, Sac1.1, lrc.51, and otk.16 (Figs. 2, 3), effectively killed mosquitoes, with lrc.51 inducing 100% larval mortality. As with bacteria [23], the larvicides retained full activity after the yeast was heat-killed (Fig. 2), which circumvents concerns for the introduction of live genetically modified organisms for mosquito control.

*Saccharomyces cerevisiae* has been cultivated worldwide for thousands of years, and this technology can be adapted to resource-limited countries with constrained infrastructures. Moreover, dried yeast can be packaged and shipped in both active (live) or inactive (dead) forms, which will facilitate regional distribution. Yeast production is readily scaled to industry-sized cultures, and so commercialization of this intervention is feasible. *S. cerevisiae*, which is non-toxic to humans, is already used globally in food and alcoholic beverage preparation. Dried inactive yeast is sold commercially as a dietary supplement and is available in tablet or flake formulations. It is anticipated that heat-inactivated yeast interfering RNA, which demonstrated high larvicidal activities in this investigation, could be prepared in bulk and distributed in these ready-to-use dried formulations.

Given the many benefits of the yeast system, the encouraging results reported in this study, as well as a recent study in the agricultural pest *Drosophila suzukii* [48], support the pursuit of proof-of-concept semi-field evaluation of yeast interfering RNA larvicides. To this end, semi-field trials with heat inactivated yeast interfering RNA larvicides are planned. This critical next phase of the project will facilitate evaluation of the efficacy, feasibility, and acceptance of introducing biorational yeast interfering RNA larvicides into integrated vector mosquito control programmes. Given that yeast, a strong odorant attractant for larvae, can act as a larval bait [10], it is likely that yeast interfering RNA pellets could be used to treat volumes of water that are much larger than those used in the present investigation, and this will need to be assessed in the field. It would also be interesting to test additional yeast formulations in the field. For example, it is possible that some species of *Anopheles* larvae may more readily ingest yeast interfering RNA flakes that float at the water surface, and this could become critical in the field, where competing food sources are available to larvae. Finally, in addition to assessing the impacts of yeast interfering RNA larvicides on the densities of juvenile and adult mosquitoes in the field, it will be important to examine the best settings for the use of these larvicides, if they can be used for control of other malaria vector mosquitoes, and ultimately to demonstrate that the larvicides can effectively reduce the number of malaria cases.

## Conclusions

In summary, new vector control tools to address residual malaria transmission are vitally needed. The laboratory trials conducted in this investigation demonstrated that yeast expressing shRNA corresponding to *An. gambiae* larval lethal genes induce up to 100% larval mortality. These yeast interfering RNA larvicides retain larvicidal activity following drying and heat-inactivation of the yeast into ready-to-use tablet formulations. Dried inactivated yeast interfering RNA larvicide tablets could someday be an effective, inexpensive, biorational addition to malaria mosquito control programmes and a valuable tool for combating residual malaria transmission.

## Additional files

**Additional file 1.** Lack of siRNA larvicide target site conservation in non‐target organisms. Summary of Blast search results.

**Additional file 2.** A lack of Sac1.1 and otk.16 yeast interfering larvicide activity in *A. aegypti* larvae. Graph depicting results from larvicide trials that demonstrated a lack of larvicidal activity for yeast interfering RNA larvicides Sac1.1 and otk.16 in *A. aegypti* larvae.

## Abbreviations

ANOVA: analysis of variance
BLAST: basic local alignment search tool
DDT: dichlorodiphenyltrichloroethane
dsRNA: double stranded RNA
EPA: Environmental Protection Agency
L1: first instar
IRS: indoor residual spraying
ITNs: insecticide treated nets
*lrc*: *leukocyte receptor complex member*
*otk*: *offtrack*
RNAi: RNA interference
shRNA: short hairpin RNA
siRNA: small interfering RNA
*Sac1*: *suppressor of actin*
WHO: World Health Organization
