# Supplementary material for: Yeast interfering RNA larvicides targeting neural genes induce high rates of Anopheles larval mortality
Source: Malar J. 2017 Nov 13;16:461. doi: 10.1186/s12936-017-2112-5 (PMC5683233; doi:10.1186/s12936-017-2112-5)
Supplement: Supplementary file 1 — Additional file 1. Lack of siRNA larvicide target site conservation in non‐target organisms. Summary of Blast search results. [file 12936_2017_2112_MOESM1_ESM.pdf]

Lack of siRNA Larvicide Target Site Conservation in Non-target Organisms

Organism or Group of Organisms	Identifying Code TaxID	Match with Sac1.1?	Match with Sac1.91?	Match with Irc.2?	Match with Irc.51?	Match with otk.16?	Match with otk.94?	Match with Control?
A. gambiae	7165	yes	yes	yes	yes	yes	yes	no
A. aegypti	7158	no	no	no	no	no	no	no
Amphibians	8292	no	no	no	no	no	no	no
Birds	8782	no	no	no	no	no	no	no
Fish	7898	no	no	no	no	no	no	no
Fungi	4751	no	no	no	no	no	no	no
Human	9606	no	no	no	no	no	no	no
Mammals	40674	no	no	no	no	no	no	no
Plants	3193	no	no	no	no	no	no	no
Reptiles	8504	no	no	no	no	no	no	no

Additional File 1. The 25 bp target sequences of the six interfering RNAs (Sac1.1, Sac1.91, Irc.2, Irc.51, otk.16, otk.94) used to silence *A. gambiae* genes in this investigation, as well as the control target sequence, were used as query sequences in NCBI BLAST searches [17] conducted using the nucleotide collection database by organism using the taxonomic identification numbers (TaxIDs) indicated. As of September 2017, other than the matches in *A. gambiae,* the organisms and taxa noted above lack identical 25 bp matches to the target sequences. Furthermore, with one exception, an identical match for the Sac1.1 target sequence in *Chlamydomonas reinhardtii* sequence XM_001692925.1, searches against all sequences in the BLAST database did not uncover any perfect matches outside of *Anopheles.*
