# Supplementary material for: Yeast interfering RNA larvicides targeting neural genes induce high rates of Anopheles larval mortality
Source: Malar J. 2017 Nov 13;16:461. doi: 10.1186/s12936-017-2112-5 (PMC5683233; doi:10.1186/s12936-017-2112-5)
Supplement: Supplementary file 2 — Additional file 2. A lack of Sac1.1 and otk.16 yeast interfering larvicide activity in A. aegypti larvae. Graph depicting results from larvicide trials that demonstrated a lack of larvicidal activity for yeast interfering RNA larvicides Sac1.1 and otk.16 in A. aegypti larvae. [file 12936_2017_2112_MOESM2_ESM.pdf]

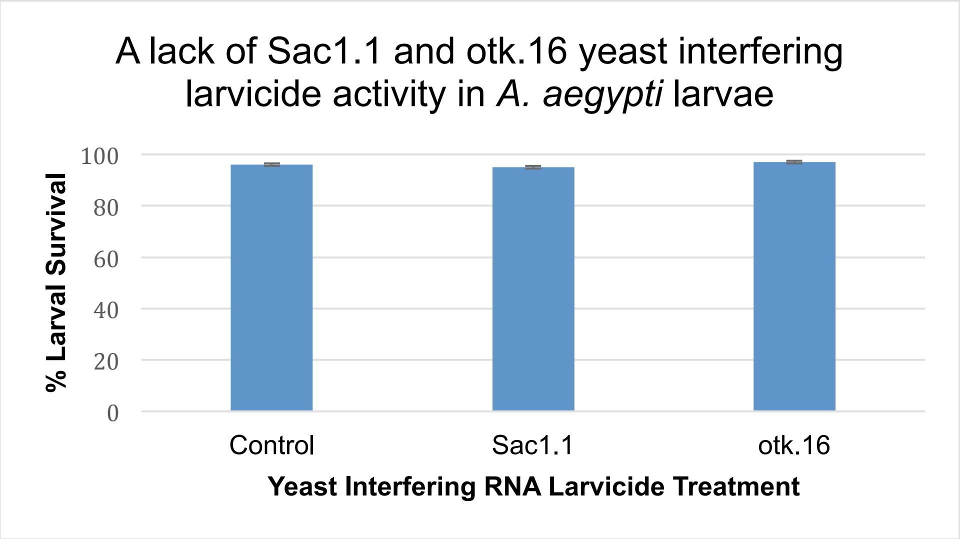

Additional File 2. *Anopheles* yeast interfering RNA larvicides do not kill *A. aegypti* larvae. No significant larval death was observed in *A. aegypti* larvae fed with yeast interfering RNA larvicides Sac1.1 or otk.16. The larvicidal activity of Sac1.1 and otk.16 yeast used in these *A. aegypti* trials was confirmed in *A. gambiae* (see text). Data were compiled from nine replicates (total n = 180 larvae/condition) and analyzed by ANOVA. ($p=0.66$ in comparison to control-fed larvae; error bars denote SEMs). These data support the species-specificity of these yeast interfering RNA larvicides.
